# IncA/C Plasmid-Mediated Spread of CMY-2 in Multidrug-Resistant *Escherichia coli* from Food Animals in China

**DOI:** 10.1371/journal.pone.0096738

**Published:** 2014-05-09

**Authors:** Yu-Fang Guo, Wen-Hui Zhang, Si-Qi Ren, Lin Yang, Dian-Hong Lü, Zhen-Ling Zeng, Ya-Hong Liu, Hong-Xia Jiang

**Affiliations:** 1 College of Veterinary Medicine, Guangdong Provincial Key Laboratory of Veterinary Pharmaceutics Development and Safety Evaluation, South China Agricultural University (SCAU), Guangzhou, China; 2 Laboratory of Clinical Microbiology, Institute of Veterinary Medicine, Guangdong Academy of Agriculture Sciences, Guangzhou, China; Institut National de la Recherche Agronomique, France

## Abstract

**Objectives:**

To obtain a broad molecular epidemiological characterization of plasmid-mediated AmpC β-lactamase CMY-2 in *Escherichia coli* isolates from food animals in China.

**Methods:**

A total of 1083 *E. coli* isolates from feces, viscera, blood, drinking water, and sub-surface soil were examined for the presence of CMY-2 β-lactamases. CMY-2-producing isolates were characterized as follows: the *bla*_CMY-2_ genotype was determined using PCR and sequencing, characterization of the *bla*_CMY-2_ genetic environment, plasmid sizing using S1 nuclease pulsed-field gel electrophoresis (PFGE), PCR-based replicon typing, phylogenetic grouping, *Xba*I-PFGE, and multi-locus sequence typing (MLST).

**Results:**

All 31 CMY-2 producers were only detected in feces, and presented with multidrug resistant phenotypes. All CMY-2 strains also co-harbored genes conferring resistance to other antimicrobials, including extended spectrum β-lactamases genes (*bla*_CTX-M-14_ or *bla*_CTX-M-55_), plasmid-mediated quinolone resistance determinants (*qnr*, *oqxA*, and *aac-(6′)-Ib-cr*), *floR* and *rmtB*. The co-transferring of *bla*_CMY-2_ with *qnrS1* and *floR* (alone and together) was mainly driven by the Inc A/C type plasmid, with sizes of 160 or 200 kb. Gene cassette arrays inserted in the class 1 or class 2 integron were amplified among 12 CMY-2 producers. CMY-2 producers belonged to avirulent groups B1 (*n* = 12) and A (*n* = 11), and virulent group D (*n* = 8). There was a good correlation between phylogenetic groups and sequence types (ST). Twenty-four STs were identified, of which the ST complexes (STC) 101/B1 (*n* = 6), STC10/A (*n* = 5), and STC155/B1 (*n* = 3) were dominant.

**Conclusions:**

CMY-2 is the dominant AmpC β-lactamase in food animals and is associated with a transferable replicon IncA/C plasmid in the STC101, STC10, and STC155 strains.

## Introduction

The prevalence of plasmid-encoded AmpC (pAmpC) β-lactamases, which confer resistance to extended-spectrum cephalosporins in Gram-negative bacilli, has increased in both humans and livestock isolates worldwide, and is a global problem [1]. CMY-2 is the most common pAmpC in *E. coli* from different geographical areas including Asia, North America, and Europe [2]–[5], and has now been reported in *Salmonella* and *Escherichia coli* isolates from a variety of food animals and products on all continents except Australia [6]. Some studies reported that the increased incidence of infections in humans with *S. enterica* serovar Newport possessing CMY-2 in North America was associated with exposure to dairy cattle as well as the consumption of raw milk, raw or improperly cooked beef mince, and the cross-contamination of raw meat with other foods [7], [8]. CMY-2-bearing plasmids, predominantly the A/C, I1, or K/B replicon types [9], [10], were readily transferable between *Salmonella* and *E. coli* from food animals and humans [10], [11]. Therefore, livestock-associated plasmid-encoded CMY-2 has posed increasing concerns to public health worldwide^6^.

*E. coli* is one of most common pathogens of nosocomial, healthcare-associated, and community infections [12]. According to CHINET (antimicrobial resistance surveillance networks in China), bacteria isolated from various samples from 14 hospitals in 10 regions or Provinces were predominantly *E. coli*, comprising 61.8% of the total isolates [13]. CMY-2 AmpC in Chinese pediatric patients was detected first in *E.coli* between 2003 and 2005; the occurrence of AmpC β-lactamase in *E. coli* and *K. pneumoniae* had the highest prevalence [13]. The resistance to multiple drugs, including third-generation cephalosporins, and the carriage of both pAmpC and extended spectrum β-lactamase (ESBL) genes in *E. coli* isolates from food animals has increased rapidly [14]-[17]. The detection rate of *bla*_CMY-2_ in isolates from chickens, which emerged between 2000 and 2003, increased rapidly from 2004–2007 [16]. However, limited information is available regarding the characteristics of the CMY-2 plasmid type or the clonal dissemination of CMY-2 in *E.coli* of food animal origin in China. The aim of this study was to assess the molecular epidemiology and characteristics of CMY-2-bearing plasmid-producing *E. coli* in food animals including pigs, chickens, ducks, and geese.

## Materials and Methods

### Bacterial isolates and antimicrobial susceptibility

A total of 1083 unique *E. coli* isolates from a range of food animal species (pigs, *n* = 424; chickens, *n* = 306; ducks, *n* = 175; geese, *n* = 178) were recovered between October 2010 and January 2012 from 58 fixed farms described previously [15]. Of the 1083 *E. coli* isolates, 587 were cultured from feces, 456 from the viscera, 14 from blood samples, and 26 from drinking water and sub-surface soil of duck farms. These isolates were collected partially from the Guangdong Province Surveillance Program on Antibiotic Resistance in bacteria isolated from animals. The program was carried out by the Laboratory of Clinical Microbiology, Veterinary Research Institute, Guangdong Academy of Agricultural Sciences. Methods for sample collection and bacterial isolation were described previously [18]. Susceptibility testing was determined for all isolates using the standard agar dilution method on Mueller-Hinton agar according to the Clinical and Laboratory Standards Institute (CLSI) guidelines [19], [20]. The 13 antimicrobials tested were ampicillin (Amp), ceftiofur (Cft), cefotaxime (Ctx), ceftazidime (Caz), cefoxitin (Cxt), ceftriaxone (Ctr), gentamicin (Gen), kanamycin (Kan), amikacin (Ami), florfenicol (Flf), tetracycline (Tet), ciprofloxacin (Cip), and olaquindox (Oqx). Cefoxitin-resistant strains (MIC ≥8 mg/L) were used for selecting CMY-2-producing strains via PCR amplification of the *bla*_CMY_ gene and DNA sequencing, molecular characterization of drug resistance, and epidemiology of *bla*_CMY-2_-harboring strains. *E. coli* ATCC 25922 was used as the control strain.

### Molecular characterization of drug resistance

Among the *bla*_CMY_-harboring strains, β-lactamase genes (*bla*_CTX-M_, *bla*_OXA_, *bla*_SHV_, *bla*_TEM_) and plasmid-mediated quinolone resistance (PMQR) genes (*qnrABCDS*, *aac(6′)-Ib-cr*, *qepA*, *oqxA*) were detected by PCR amplification using specific primers and conditions (Table S1). The 16S rRNA methylase genes *rmtB* and the plasmid-borne florfenicol resistance gene, *floR*, were similarly detected using primers listed in Table S1.

### Genetic environment of *bla*_CMY-2_ gene and detection of integrons

The genetic environment of the *bla*_CMY-2_ genes was investigated using PCR and sequencing. The IS*Ecp1* forward primer and CMY-2 reverse primer were used to investigate regions upstream of the *bla*_CMY-2_ genes. The CMY-2 forward primer and reverse primers for IS*903*, IS*26*, *orf447*, *mucA*, or *orf513* were used to characterize regions downstream of the *bla*_CMY-2_ genes. Integrons of class 1 or class 2 as well inserted gene cassettes were detected. Sequences of the above primers are listed in Tables S2 and S3.

### Conjugation and plasmid analysis

Conjugation experiments were performed on *bla*_CMY-2_-containing strains using streptomycin-resistant *E. coli* C600 as the recipient [4]. Transconjugants were selected on MacConkey agar plates supplemented with streptomycin (1000 mg/L) and cefoxitin (8 mg/L). Transconjugants were tested for the presence of drug resistance genes and antimicrobial susceptibility, as described above.

Plasmids were typed using PCR-based replicon typing (PBRT) [21]. PFGE with S1 nuclease (TakaRa Biotechnology, Dalian, China) digestion of whole genomic DNA was performed for all 15 donor strains and transconjugants, as described previously [22]. After Southern transfer to a Hybond-N^+^ membrane (GE Healthcare, Little Chalfont, United Kingdom), the plasmids were probed with the *bla*_CMY-2_ gene and respective replicons (DIG High Prime DNA Labeling and Detection Starter Kit I, Roche Applied Science, Mannheim, Germany).

### Population structure analysis

All CMY-2-producing *E. coli* isolates were classified according to *E. coli* phylogenetic groups A, B1, B2, and D using multiplex PCR [23]. *Xba*I-pulsed-field gel electrophoresis (PFGE) patterns were typed using a CHEF-MAPPER System (Bio-Rad Laboratories, Hercules, CA) as described previously [15]. For multi-locus sequence typing (MLST) analysis, seven conserved housekeeping genes (*adk*, *fumC*, *gyrB*, *icd*, *purA*, *mdh*, and *recA*) were analyzed by PCR amplification using specific primers (Table S4) and sequencing. Allelic profiles and sequence type (ST) determinations were performed according to the *E. coli* MLST website (http://mlst.ucc.ie/mlst/dbs/E.coli) scheme.

## Results

### Antimicrobial susceptibility

A total of 233 (21.5%) out of 1083 *E. coli* isolates were resistant to cefoxitin with MIC values ranging from 8 mg/L to >512 mg/L. Only 33 of the cefoxitin-resistant isolates produced CMY, including 19 from pigs, 10 from chickens, and two each from ducks and geese. Of the 33 *bla*_CMY_ bearing isolates, 31 carried *bla*_CMY-2_ from feces (Table 1), and two from the feces and viscera of geese carried *bla*_CMY-41_ and *bla*_CMY-64_, respectively (data not shown). All 33 CMY-producing isolates exhibited multi-drug resistance profiles, and showed resistance to both β-lactam drugs and more than two non-β-lactam drugs. The most common resistance pattern was Amp-Ctx-Caz-Ctr-Flf-Gen-Kan-Tet-Cip-Oqx (20/33, 61%). The presence of resistance to olaquindox (a growth promoter used extensively in pig and poultry farms) was detected in the majority of *bla*_CMY-2_-bearing strains (28/33, 85%). Twenty-three *bla*_CMY-2_-bearing isolates were resistant to ceftiofur, a newly approved β-lactam for veterinary use in China. Resistance to amikacin was relatively low (8/32, 25%).

**Table 1:** Overall results of co-resistance, phylogenetic grouping, MLST and plasmid replicon analysis of CMY-2producing E. coli isolates of food producing animals in China.

Strains	Source	Other resistance genes	Phylogen. group	MLST		*bla_CMY-2_* linked-element upstream	Gene cassettes inserted in class1 and 2 integrons	Plasmid transfer	Co-transferred resistant gene	Plasmid replicon types and approx. size (kb)
				ST	STC^a^					
L145	Pig	*bla_OXA-1_*,*oqxA*,*floR*	A	10	STC10	IS*Ecp1*	ND	-	-	-
L147	Pig	*oqxA*	A	10	STC10	IS*Ecp1*	ND	-	-	-
L393	Chicken	*bla_TEM-1_*,*qnrS1*,*oqxA*,*floR*,*aac-(6′)-Ib-cr*	A	2690	STC10	IS*Ecp1*	ND	+	*qnrS1*,*floR*,*aac-(6′)-Ib-cr*	A/C,160
L699	Chicken	*bla_TEM-1_*,*qnrS1*,*floR*	A	48	STC10	IS*Ecp1*	class2: *sat1*+*aadA1*	+	*qnrS1*,*floR*	A/C,200
L78	Pig	*bla_CTX-M-14_*,*bla_TEM-1_*,*qnrS1*,*oqxA*,*floR*	A	3244	STC10	IS*Ecp1*	ND	+	*bla_TEM-1_*,*qnrS1*,*floR*	A/C,160
L667	Pig	*bla_TEM-1_*,*oqxA*	B1	3403	STC101	IS*Ecp1*	class1: *drfA17*+*aadA5*	-	-	-
L671	Pig	*bla_TEM-1_*,*bla_OXA-1_*,*oqxA*,*qnrS1*	B1	359	STC101	IS*Ecp1*	class1: *drfA17*+*aadA5*	+	*qnrS1*	K,200
L669	Pig	*bla_TEM-1_*,*oqxA*	B1	359	STC101	IS*Ecp1*	class1: *drfA17*+*aadA5*	-	-	-
L670	Pig	*bla_TEM-1_*,*oqxA*,*aac-(6′)-Ib-cr*	B1	359	STC101	IS*Ecp1*	class1: *drfA17*+*aadA5*	-	-	-
L679	Pig	*bla_TEM-1_*,*qnrS1*,*oqxA*,*floR*	B1	101	STC101	IS*Ecp1*	ND	-	-	-
L1119	Pig	*bla_TEM-1_*,*qnrS1*,*aac-(6′)-Ib-cr*,*oqxA*,*floR*	B1	101	STC101	IS*Ecp1*	ND	+	*qnrS1*,*floR*	A/C,160
L518	Pig	*bla_TEM-1_*,*oqxA*,*rmtB*	D	648	none	IS*Ecp1*	ND	-	-	-
L461	Duck	*bla_CTX-M-55_*,*bla_TEM-1_*,*qnrS1*,*oqxA*,*floR*,*rmtB*	D	648	none	IS*Ecp1*	class1: *aadA22*	+	*bla_CTX-M-55_*, *qnrS1*	FIB,40
L351	Chicken	*floR*,*rmtB*	B1	155	ST155C	IS*Ecp1*	ND	+	*floR*	A/C,200
L391	Chicken	*bla_TEM-1_*,*qnrS1*,*floR*	B1	155	ST155C	IS*Ecp1*	class1*: dfrA1*+*aadA1*	+	*qnrS1*,*floR*	A/C,160
L392	Chicken	*bla_TEM-1_*,*qnrB6*,*floR*	B1	2294	ST155C	IS*Ecp1*	class1: *dfrA1*+*aadA1*	+	*floR*	A/C,160
L813	Pig	*bla_TEM-1_*,*qnrS1*,*rmtB*,*aac-(6′)-Ib-cr*	B1	156	none	IS*Ecp1*	ND	+	*qnrS1*	FIB,40
L361	Duck	*bla_TEM-1_*,*qnrS1*,*aac-(6′)-Ib-cr*	D	156	none	IS*Ecp1*	ND	-	-	-
T117	Pig	*bla_TEM-1_*	A	1114	none	IS*Ecp1*	ND	-	-	-
A10-2	Pig	*bla_TEM-1_*,*bla_OXA-1_*,*qnrS1*,*oqxA*,*floR*	A	1114	none	IS*Ecp1*	ND	-	-	-
L1039	Pig	*bla_TEM-1_*,*oqxA*,*floR*	D	457	ST457C	IS*Ecp1*	ND	-	-	-
T43	Pig	*oqxA*	D	3376	ST457C	ND	ND	-	-	-
C42	Pig	*floR*	A	3402	none	IS*Ecp1*	ND	-	-	-
T26	Pig	*bla_TEM-1_*,*oqxA*,*floR*	A	3404	none	ND	ND	+	*bla_TEM-1_*,*floR*	A/C,160
L1166	Pig	*floR*	A	3269	none	IS*Ecp1*	ND	+	*floR*	A/C,160
L653	Pig	*bla_TEM-1_*,*oqxA*,*floR*	A	3014	none	IS*Ecp1*	ND	-	-	-
L215	Chicken	*bla_TEM-1_*	D	362	none	IS*Ecp1*	class1: *drfA17*+*aadA5*	-	-	-
L349	Chicken	*qnrS1*,*floR*	D	354	none	IS*Ecp1*	class1: *drfA17*+*aadA5*	+	*qnrS1*,*floR*	A/C,200
L394	Chicken	*qnrS1*,*bla_TEM-1_*,*floR*	B1	3245	none	IS*Ecp1*	class1: *orf*+*aadA2*	+	*bla_TEM-1_*,*qnrS1*,*floR*	HI2,220
L398	Chicken	-	B1	1431	none	ND	class1: *dfrA17*+*aadA5*	-	-	-
L399	Chicken	*qnrS1*	D	69	none	IS*Ecp1*	ND	+	*qnrS1*	K,160

a STC, ST complex.

ND, not detected.

### Characterization of *bla*_CMY-2_ harboring isolates

All except one CMY-2-producing isolate harbored more than one resistance gene conferring resistance to different antimicrobial drugs (Table 1). Of the detected ESBLs genes, *bla*_CTX-M_ type alone (*bla*_CTX-M-14_ and *bla*_CTX-M-55_) was identified in two strains; no isolate harbored the *bla*_SHV_ gene. The narrow β-lactamase-encoding genes *bla*_TEM-1_ and *bla*_OXA-1_ were found in 22 and three strains, respectively. Among the detected PMQR determinants, *oqxA*, *qnr*, and *aac-(6′)-Ib-cr* were detected in 17, 15, and five strains, respectively (Table 1). Of the 15 *qnr* genes, 14 were *qnrS1*, and one was *qnrB6*. No *qnrA, qnrD, qnrC*, or *qepA* genes were detected. The *floR* and *rmtB* genes were identified in 18 and four strains, respectively. Both ESBL producers also carried the *qnrS1*, *oqxA*, and *floR* genes. Remarkably, one strain (L461) contained five genes conferring resistance to five antimicrobial drug classes (Table 1). A CMY-41 producer from goose liver concurrently carried *bla*_CTX-M-15_ and *bla*_CTX-M-65_ (data not shown).

### Genetic environment of *bla*_CMY-2_ and detection of integrons

Twenty-eight of 31 *bla*_CMY-2_ were linked to an upstream IS*Ecp1* element; *bla*_CMY-41_ was also associated with the upstream IS*Ecp1* element. No IS elements were detected upstream of *bla*_CMY-64_ or downstream of *bla*_CMY_. Of the 31 CMY-2 producers, 27 contained class 1 integrases, and one harbored a class 2 integrase. Of the 27 class 1 integrase-positive isolates examined, 11 were found to possess cassettes inserted within the integrons, including the four-gene cassette arrays *dfrA17*+*aadA5*, *dfrA1*+*aadA1*, *orfF*+*aadA2*, and *aadA22* (Table 1). *dfrA17*+*aadA5* was most common (7/11, 64%), followed by *dfrA1*+*aadA1* (2/11, 18%); *orfF*+*aadA2* and *aadA22* were found in single isolates. The class 2 integron present in one strain (L699) harbored the 1.0 kb *sat1*+*aadA1* arrays. One CMY-41 producer from geese also contained a *dfrA17*+*aadA5* cassette array.

### Population structure analysis

All 31 *bla_CMY-2_* strains were distributed into groups B1 (*n* = 12) and A (*n* = 11) of the commensal strains, strains associated with enterotoxigenic and enterohemorrhagic infections, and the potentially virulent phylogenetic group D (*n* = 7). The strain carrying *bla*_CMY-41_ from the liver of a diseased goose belonged to group D, and the strain containing *bla*_CMY-64_ from a goose intestine belonged to group A.

**Figure 1:**
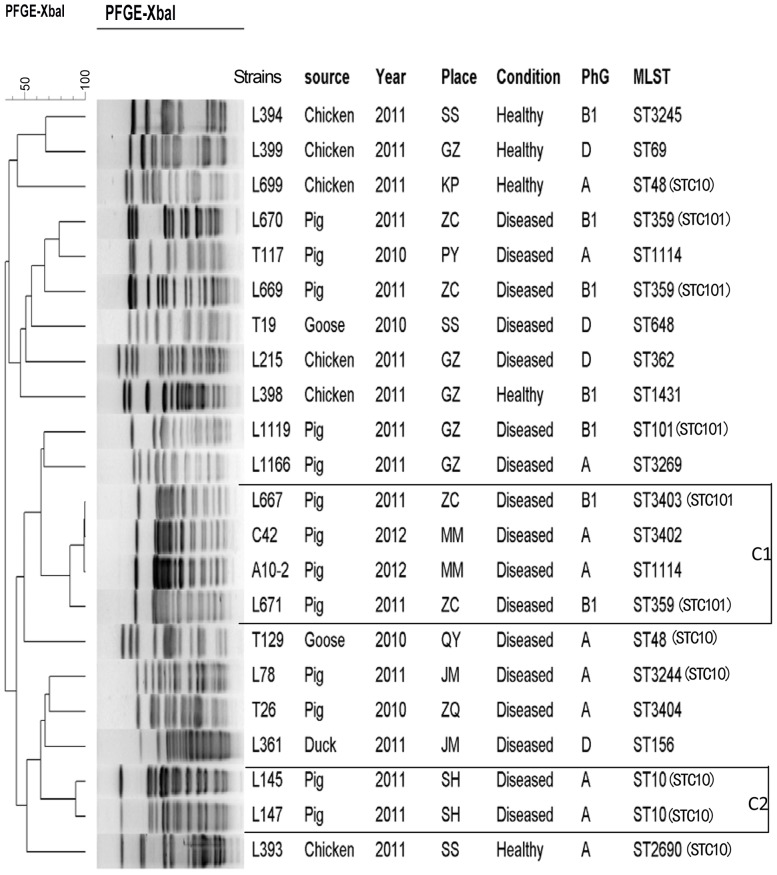
Dendrogram of *Xba*I-PFGE patterns of CMY-producing *E. coli* strains recovered from food-producing animals. All the strains were CMY-2 producers, except for T19 (CMY-41) and T129 (CMY-64). Similarity analysis was performed using the Dice coefficient, and clustering was performed by following the unweighted-pair group method using average linkages (UGPMA). A total of 16 PFGE patterns were identified, and the two clusters with highly similar PFGE patterns were labeled C1 and C2. Abbreviations for the place column: SS, Sanshui; GZ, Guangzhou; KP, Kaiping; ZC, Zengcheng; PY, Panyu; MM, Maoming; QY, Qingyuan; JM, Jiangmen; ZQ, Zhaoqing; SH, Sihui. Abbreviation for PhG: Phylogenetic Group.

A total of 18 different PFGE-types were detected among 22 typeable CMY isolates (20 CMY-2, one CMY-41, and one CMY-64), including 19 single types, and two clusters (>90% similarity) containing two (Cluster 2) and four isolates each (Cluster1) (Fig. 1). Interestingly, the four isolates in Cluster1 were obtained from two different locations and further divided into two phylogenetic groups, each containing two isolates. The Cluster 2 isolates belonged to the same phylogenetic group and originated from the same place.

MLST analysis of the 31 CMY-2 producing isolates identified 24 different STs, including seven novel ones (ST3244, ST3245, ST3403, ST3269, ST3402, ST3404, and ST3376) (Table S5). A total of 20 isolates were related to six STs or ST-complexes (STCs) described previously: 10, 101, 155, 156, 648, and 1114. The prevalent ST/STCs were 101 (*n* = 6, 19%), 10 (*n* = 5, 16%), 155 (*n* = 3, 10%), 156 (*n* = 2, 6%), 457 (*n* = 2, 6%), 1114 (*n* = 2, 6%), and ST648 (*n* = 2, 6%). The remaining isolates were each of a single ST type, including four novel ST types (Table S5).

STC 101 included ST101, the single locus variant (SLV; isolate ST359), and one double locus variant (DLV; isolate ST3403). STC 10 included ST10, one SLV isolate (ST48), one DLV isolate (ST2690), and one triple locus variant (TLV) isolate (the novel ST3244). STC155 included two ST155 and one SLV isolate (ST2294).

### Transferability of *bla*_CMY-2_ and plasmid analysis

The transferability of *bla*_CMY-2_ was observed in 15 out of 31 isolates at transfer frequencies ranging from 10^−3^ to 10^−7^ transconjugants per recipient. Of the 15 transferable *bla*_CMY-2_, 10 were located on the IncA/C type plasmid with sizes of 160 to 200 kb, two 40 kb IncFIB plasmids, two IncK plasmids sized 160 kb and 200 kb, and one 220 kb HI2 plasmid. Genes encoding resistance to other antimicrobials also co-transferred in some strains. Remarkably, *qnrS1* and *floR* co-transferred with *bla*_CMY-2_ in eight of 15 isolates. The co-transfer of *bla*_CMY-2_, *bla*_CTX-M-55_, and *qnrS1* was observed in one isolate from a duck.

## Discussion

In the present study, we performed a broad molecular epidemiological characterization of CMY-2-producing *E. coli* collected from the Guangdong Province Surveillance Program on antibiotic resistance in bacteria isolated from animals between 2010 and 2012. Compared with earlier surveillance on *E. coli* antibiotic resistance from fixed food animal farms, resistance to a panel of cephalosporins was increased significantly from <7% to between 20 and 60% (data not shown) for all tested drugs in 2003 to 2005 [14], [15]; resistance to cefotaxime, ceftriaxone, cefoxitin, ceftiofur, and ceftazidime was 55.8, 59.7, 21.5, 60, and 20.8%, respectively. The rapid increase in resistance to third-generation cephalosporins, particularly for ceftiofur, which is newly approved for use in veterinary clinical settings, was consistent with previous reports from other Provinces in China [16], [17].

Cefoxitin/ceftiofur resistant isolates from *E. coli* and *Salmonella* occur frequently worldwide, which is likely to be associated with the production of CMY enzyme(s) [24]. The use of ceftiofur in the veterinary clinical setting has encouraged selection of CMY-2 AmpC in both *Salmonella* and *E. coli* [6]. In China, the occurrence of CMY-2 in *E. coli* originating in chickens increased rapidly [16]; the detection rate of *bla*_CMY-2_ in chicken isolates was higher than in those from pigs [25], which is consistent with reports from Japan [26]. In the present study, all CMY-2 producers were isolated from feces, consistent with a previous study [14], suggesting that the gastro-intestinal tract of animals is a reservoir for CMY-2 producers. Only half of the samples were collected from animal feces in the present study, which might explain the lower occurrence of CMY-2. Of note, many CMY-2 producers were isolated from pigs, which was an increased prevalence compared with previous reports [14], [25]. The use of ceftiofur was approved for pigs in 2005 in China, which may have contributed to this rapid increase.

*Salmonella* and *E. coli* isolates carrying *bla*_CMY-2_ have been associated with community-acquired infections [1], [6], [27]. Plasmids carrying AmpC genes often carry other genes that confer resistance to non-β-lactams, but rarely ESBL genes [28]. Previous studies demonstrated that CMY-2-producing *E. coli* easily acquired other resistance genes, resulting in a multidrug resistant profile. The *floR* gene was the most common gene acquired, which conferred resistance to florfenicol. Some studies have shown that the increasing occurrence of CMY-2 was associated with the use of florfenicol in the veterinary clinic [29]. In the present study, most CMY-2 producers harbored not only genes encoding β-lactamases (including ESBLs), but also many diverse genes encoding resistance to other antimicrobials. In addition to *floR*, variants of *qnr*, a plasmid-mediated quinolone resistant gene, were detected frequently. The presence of *qnr* or *floR*, either alone or in combination, was detected frequently in CMY-2 isolates. Transconjugation experiments confirmed that the *bla*_CMY-2_ gene could co-transfer with multiple antibiotic resistance genes, frequently with *qnrS1*+*floR* or *qnrS1*, driven by 160 and 200 kb-sized IncA/C plasmids. Previous studies demonstrated that plasmids carrying CMY-2 could self-transfer between different strains alone, but rarely co-transferred with multiple genes [4], [27], [30]. The spread of *bla*_CMY-2_ was driven mainly by the IncA/C, IncI1, or IncK plasmids [31].

Co-localization of *bla*_CMY-2_ and *floR* on an IncA/C plasmid was detected commonly. A close relationship between the IncA/C plasmid and the multidrug resistance (MDR) of plasmid bearing isolates from human, animal, and environmental origins has been reported [9]. Considering the high occurrence of MDR genes (such as *floR* and *qnr*) in IncA/C plasmids and the co-transfer of these genes within the same plasmids in the present study, we suggest that there should be a stable association of these resistance genes with A/C plasmids. In addition, *bla*_CMY-2_ co-transferred with *qnrS1* located on the IncK plasmid, with sizes of 160 and 200 kb. These results highlight the potential risk for co-selection of isolates carrying *bla*_CMY-2_ via the use of florfenicol or fluoroquinolones in the raising of food animals [32], [33]. Interestingly, the co-transfer of *bla*_CMY-2_ with *qnrS1*+*floR* located on the IncHI2 plasmid was first reported in a chicken isolate. The IncHI2 plasmid was most prevalent in *Salmonella* isolates of food animal origin, and could readily capture PMQR [34]. Our results suggest that the dissemination of IncHI2 plasmids carrying CMY-2, PMQR, and *floR* between *E. coli* and *Salmonella* might have occurred.

Although the occurrence of OqxAB was surprisingly high in China (39%) [35] compared with Denmark (1.8%), and Korea (0.4%) [36], [37], the encoded genes rarely co-transferred with *bla_CMY-2_* on the same plasmid. Previous findings demonstrated that *rmtB* was the most prevalent 16S rRNA methylase gene in the *Enterobacteriaceae* isolates that produced ESBLs in China [23], [38]. Similarly, only the *rmtB* gene was detected in five strains in this study. However, in contrast with the previously described co-transfer of *rmtB* and ESBL-encoding genes, the co-transfer of *rmtB* with *bla*_CMY-2_ was not observed in the current study, as demonstrated by a conjugation experiment.

Plasmids and integrons can contain or capture a variety of resistance genes that are beneficial for survival of the bacterial host and help them adapt to changing environments [39], [40]. Integrons are genetic platforms involved in the spread of different previously captured gene cassettes that encode determinants of antimicrobial-resistance and represent a fundamental resource for bacterial evolution [41]. Integrons are divided into five classes based on integrase gene sequence; class 1 integrons are by far the most common in clinical isolates of Gram-negative bacteria [41]. In this study, we found five types of integrons encompassing eight different genes: *aadA1*, *aadA2*, *aadA5*, *aadA22*, *dfrA1*, *dfrA17*, *sat1*, and *orfF*. The most common integron profile (*dfrA17*-*aadA5*) was found in *Salmonella* and other *Enterobacteriaceae*, and recently in two *Staphylococcus* species isolated in China, suggesting the successful spread of this integron around the world and across bacterial phyla [40]. Different types of integrons contained by CMY-2 producers represented a diverse trend in strain evolution. DNA fingerprints also revealed that most CMY-2 producers were unlikely to be derived from a single *E. coli* clone. However, the clonal dissemination of *bla*_CMY-2_ between different farms at the same and different geographical locations was also found.

There was strong correlation between phylogenetic groups and STs in this study. One of the most common ST lineages, STC101, was isolated from pigs, and belonged to the avirulent phylogroup B1. Most of these harbored class 1 integron-containing *drfA17*+*aadA5* cassette arrays. The global spread of *bla*_CTX-M-9G_ ESBLs and *bla*_NDM-1_ was driven by ST101/B1 *E. coli*, and could be explained by the accumulation of a large number of virulence genes [42]. Similarly ST101C/B1 CMY-2, which is prevalent dominantly and consistently at pig farms from different geographical regions, could be explained by its ability for acquiring class 1 integrons with the most common cassettes arrays and antibiotic resistant genes. Capturing and acquiring these genes could help establish the dissemination of STC101/B1 CMY-2 isolates between pig farms. The other most common ST, STC10, belonged to the avirulent phylogroup A, and was distributed widely between pigs, chickens, and geese. The significant contribution of ST10C to the spread of resistance in humans was reported in Europe and Canada [43]. Interestingly, a chicken origin ST48 (belonging to ST10C) contained *qnrS1*+*floR*, as well a class 2 integron carrying a *sat1*+*aadA1* cassette array. Compared with ST10, its SLV or DLV contained more antibiotic resistant genes, which helps the host bacteria adapt to their surroundings under antibiotic selective pressures. Three strains isolated from a diverse range of animal species corresponded to ST648, which belongs to virulent phylogroup D. It was worthy noting that two of these three strains contained class 1 integrons with different cassette arrays, which might allow the potential persistence between animals. *E.coli* strains of ST648/D clones were reported to cause most cases of ESBL-producing *E. coli* bacteremia in the Netherlands [44]. Additional concerns arise from this ST belonging to phylogroup D, due to its ability to produce New Delhi metallo (NDM)-type carbapenemases in hospitalized patients in Pakistan and the United Kingdom [45], [46]. In a recent study, ST648/D clones were the main vectors that allowed the spread of *bla*_CMY-2_ between dogs in the Republic of Korea [47].

This study demonstrated the presence of *bla*_CMY-2_ in broad host-range conjugative plasmids. To our knowledge, this represents the first comprehensive analysis of CMY-2 plasmids in *E. coli* isolated from food animals in China. *bla*_CMY-2_ was co-transferred with *qnrS1* and/or *floR*, linked to diverse lineages of *E. coli* STCs (including 101, 10, and 155), and disseminated among different food producing animals in China. The acquisition of multiple antimicrobial resistant genes and integrons might have allowed CMY-2-positive isolates to persist in the environment and evolve under antibiotic selective pressure. Continued surveillance of CMY-2 in animal reservoirs is necessary to curb the spread of multidrug resistant pathogens from animals to humans.

## Supporting Information

Table S1 **Primers used for the PCR amplification of antimicrobial resistance genes.**
(DOC)

Table S2 **Primers used for the PCR amplification of genetic environment of** ***bla*** **_CMY-2_ gene.**
(DOC)

Table S3 **Primers used for the PCR amplification of integrons.**
(DOC)

Table S4 **MLST Primers used for the PCR amplification of** ***E. coli*** **.**
(DOC)

Table S5 **The number of alleles and ST results for thirty-one CMY-2 producing strains.**
(DOC)
