# Supplementary material for: IncA/C Plasmid-Mediated Spread of CMY-2 in Multidrug-Resistant Escherichia coli from Food Animals in China
Source: PLoS One. 2014 May 9;9(5):e96738. doi: 10.1371/journal.pone.0096738 (PMC4016023; doi:10.1371/journal.pone.0096738)
Supplement: Table S1 — Primers used for the PCR amplification of antimicrobial resistance genes. (DOC) [file pone.0096738.s001.doc]

**Table S1** Primers used for PCR amplification of antimicrobial resistance genes

| Gene | Sequence (5'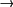3') | Amplicon size (bp) | Reference |
| --- | --- | --- | --- |
| *bla*_CMY_ | F: GACAGCCTCTTTCTCCACA | 1146 | 1 |
|  | R: TGGAACGAAGGCTACGTA |  |  |
| *bla*_CTX-M_ | F: TTTGCGATGTGCAGTACCAGTAA | 544 | 2 |
|  | R: CGATATCGTTGGTGGTGCCATA |  |  |
| *bla*_TEM_ | F: ATAAAATTCTTGAAGACGA | 1080 | 3 |
|  | R: GACAGTTACCAATGCTTAATC |  |  |
| *bla*_SHV_ | F:TTATCTCCCTGTTAGCCACC | 795 | 3 |
|  | R: GATTTGCTGATTTCGCTCGG |  |  |
| *bla*_OXA_ | F: ACACAATACATATCAACTT | 813 | 4 |
|  | R: AGTGTGTTTAGAATGGTGA |  |  |
| *qnrA* | F: ATTTCTCA CGCCAGGATTTG | 516 | 5 |
|  | R: GGCAGCACTATTACTCCCA |  |  |
| *qnrB* | F: GATCGTGAAAGCCAGAAA | 469 | 5 |
|  | R: ACGATGCCTGGTAGTTGTC |  |  |
| *qnrC* | F: GGGTTGTACATTTATTGAATC | 447 | 6 |
|  | R: TCCACTTTACGAGGTTCT |  |  |
| *qnrD* | F: CGAGATCAATTTACGGG | 582 | 7 |
|  | R: AACAAGCTAGAGCGCCTG |  |  |
| *qnrS* | F: ACGACATTCGTCAACTGC | 417 | 5 |
|  | R: TAAATTGGCACCCTGTAG |  |  |
| *qepA* | F: CGTGTTGCTGGAGTTCTTC | 403 | 8 |
|  | R: CTGCAGGTACTGCGTCATG |  |  |
| *aac(6')-Ib-cr* | F: TTGCGATGCTCTATGAGTGGCTA | 482 | 8 |
|  | R: CTCGAATGCCTGGCGTGTTT |  |  |
| *oqxA* | F:AGTCCATACCAACCTCGTCTCC | 529 | this study |
|  | R: GCGTGGCTTTGAACTCTGC |  |  |
| *rmtB* | F:ACATCAACGATGCCCTCAC | 725 | 9 |
|  | R：AAGTTCTGTTCCGATGGTC |  |  |
| *floR* | F：TTTGGWCCGCTMTCRGAC | 480 | 10 |
|  | R：SGAGAARAAGACGAAGAAG |  |  |

**References**

1. Zhao S, White DG, McDermott PF, Friedman S, English L, et al. ( 2011) Identification and expression of cephamycinase *bla*_CMY_ genes in *Escherichia coli* and *Salmonella* isolates from food animals and ground meat. Antimicrob Agents Chemother 45: 3647-3650

2. Edelstein M, Pimkin M, Palagin I, Edelstein I, Stratchounski L (2003) Prevalence and molecular epidemiology of CTX-M extended spectrum β-lactamase production in *Escherichia coli* and *Klebsiella pneumoniae* in Russian hospitals. Antimicrob Agents Chemother 47:3724−3732.

3. Weill FX, Demartin M, Tande D, Espie E, Rakotoarivony I, et al. (2004) SHV-12-like extended-spectrum-beta-lactamase-producing strains of *Salmonella enterica* serotypes Babelsberg and Enteritidis isolated in France among infants adopted from Mali. J Clin Microbiol 42: 2432-2437.

4. Briñas L, Moreno MA, Zarazaga M, Porrero C, Sáenz Y, et al. (2003) Detection of CMY-2, CTX-M-14, and SHV-12 β-Lactamases in *Escherichia coli* fecal-sample isolates from healthy chickens. Antimicrob Agents Chemother 47:2056–2058。

5. Robicsek A, Strahilevitz J, Sahm,Jacoby DF, GA, Hooper DC (2006) qnr prevalence in ceftazidime-resistant Enterobacteriaceae isolates from the United States. Antimicrob. Agents Chemother. 50:2872–2874.

6. Wang MH, Guo QG, Xu XG, Wang X, Ye X, et al. (2009) New plasmid-mediated quinolone resistance gene, qnrC, found in a clinical isolate of *Proteus mirabilis*. Antimicrob. Agents Chemother 10:1892–1897

7. Cavaco LM, Hasman H, Xia S, Aarestrup FM (2009) qnrD, a novel gene conferring transferable quinolone resistance in *Salmonella enterica* serovar Kentucky and Bovismorbificans strains of human origin. Antimicrob. Agents Chemother 10:603–608

8. Minarini LA, Poirel L, Cattoir V, Darini AL, Nordmann P (2008) Plasmid-mediated quinolone resistance determinants among enterobacterial isolates from outpatients in Brazil. J Antimicrob Chemother. 62:474-478

9. Chen L, Chen ZL, Liu JH, Zeng ZL, Ma JY, et al. (2007) Emergence of RmtB methylase-producing *Escherichia coli* and Enterobacter cloacae isolates from pigs in China. J Antimicrob Chemother 59:880-885.

10. Arcangioli MA, Leroy-Setrin S, Martel JL, Chaslus-Dancla E (1999) A new chloramphenicol and florfenicol resistance gene flanked by two integron structures in *Salmonella typhimurium DT104.* Fems Microbiol Letters 174:327-332.
