# Supplementary material for: IncA/C Plasmid-Mediated Spread of CMY-2 in Multidrug-Resistant Escherichia coli from Food Animals in China
Source: PLoS One. 2014 May 9;9(5):e96738. doi: 10.1371/journal.pone.0096738 (PMC4016023; doi:10.1371/journal.pone.0096738)
Supplement: Table S2 — Primers used for the PCR amplification of genetic environment of bla CMY-2 gene. (DOC) [file pone.0096738.s002.doc]

**Table S2** Primers used for PCR amplification of genetic environment of *bla_CMY-2_* gene

| Gene | Sequence (5'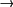3') | Reference |
| --- | --- | --- |
| IS*Ecp*1 | F:TTCAAAAAGCATAATCAAAGCC | 1 |
|  | R:CAACCACCTTTCAATCATTTTT |  |
| IS903 | R:CATCCAGCCAGAAAGTT | 1 |
| IS26 | R:CAAAGTTAGCGATGAGGCAG | 1 |
| Orf477 | R:ACT TCA AAA ATT ATG CCA CC | 1 |
| ORF513 | R:CCGTTGCACTCTCTTTGTCAC | 1 |
| mucA | GGC ATC AGG CAG GGG TAA GG | 1 |

**References**

1. Eckert C, Gautier V, Arlet G (2006) DNA sequence analysis of the genetic environment of various *bla*_CTX-M_ genes. J Antimicrob Chemother 57:14-23.
