# Supplementary material for: IncA/C Plasmid-Mediated Spread of CMY-2 in Multidrug-Resistant Escherichia coli from Food Animals in China
Source: PLoS One. 2014 May 9;9(5):e96738. doi: 10.1371/journal.pone.0096738 (PMC4016023; doi:10.1371/journal.pone.0096738)
Supplement: Table S3 — Primers used for the PCR amplification of integrons. (DOC) [file pone.0096738.s003.doc]

**Table S3** Primers used for PCR amplification of integrons

| target | Sequence (5'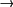3') | Amplicon size (bp) | Reference |
| --- | --- | --- | --- |
| *intI*1 | F:CCTCCCGCACGATGATC | 280bp | 1 |
|  | R:TCCACGCATCGTCAGGC |  |  |
| *intI*2 | F: TTATTGCTGGGATTAGGC | 233bp | 1 |
|  | R: ACGGCTACCCTCTGTTATC |  |  |
| *intI*3 | F: AGTGGGTGGCGAATGAGTG | 600bp | 1 |
|  | R: TGTTCTTGTATCGGCAGGTG |  |  |
| *intI*4 | F: CGGTATGTCTAATTGCTCTTG | 696bp | 1 |
|  | R:TGGCCACAAAGACTCAATCAC |  |  |
|  | sul1-R:GAAGAACCGCACAATCTCGTC |  |  |
| Class 1variable region | F: GGCATCCAAGCAGCAAG | variable | 2 |
|  | R:AAGCAGACTTGACCTGA |  |  |
| Class 2variable region | hep74:CGGGATCCCGGACGGCATGCACGATTTGTA  hep51: GATGCCATCGCAAGTACGAG | variable | 3 |

**References**

1. Goldstein C, Lee MD, Sanchez S, Hudson C, Phillips B, et al. (2001) Incidence of class 1 and 2 integrases in clinical and commensal bacteria from livestock, companion animals, and exotics. Antimicrob Agents Chemother 45:723-726

2. Lévesque C, Piché L, Larose C, Roy PH (1995) PCR Mapping of integrons reveals several novel combinations of resistance genes. Antimicrob Agents Chemother 39:185-191.

3. White PA, McIver CJ, Rawlinson WD (2001) Integrons and gene cassettes in the enterobacteriaceae. Antimicrob Agents Chemother 45:2658-2661.
