# Supplementary material for: IncA/C Plasmid-Mediated Spread of CMY-2 in Multidrug-Resistant Escherichia coli from Food Animals in China
Source: PLoS One. 2014 May 9;9(5):e96738. doi: 10.1371/journal.pone.0096738 (PMC4016023; doi:10.1371/journal.pone.0096738)
Supplement: Table S4 — MLST Primers used for the PCR amplification of E. coli . (DOC) [file pone.0096738.s004.doc]

**Table S4** MLST Primers used for PCR amplification of *E. coli*

| Gene | Sequence (5'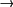3') | Amplicon size (bp)  size (bp) | Reference |
| --- | --- | --- | --- |
| *adk* | F:TCATCATCTGCACTTTCCGC | 583bp | 1 |
|  | R:CCAGATCAGCGCGAACTTCA |  |  |
| *fumC* | F:TCACAGGTCGCCAGCGCTTC | 806bp | 1 |
|  | R:TCCCGGCAGATAAGCTGTGG |  |  |
| *gyrB* | F:GTCCATGTAGGCGTTCAGGG | 911bp | 1 |
|  | R:ATCAGGCCTTCACGCGCATC |  |  |
| *icd* | F:ATGGAAAGTAAAGTAGTTGTTCCGGCACA | 878bp | 1 |
|  | R:GGACGCAGCAGGATCTGTT |  |  |
| *mdh* | F:AGCGCGTTCTGTTCAAATGC | 932bp | 2 |
|  | R:CAGGTTCAGAACTCTCTCTGT |  |  |
| *purA* | F:TCGGTAACGGTGTTGTGCTG | 816bp | 1 |
|  | R:CATACGGTAAGCCACGCAGA |  |  |
| *recA* | F:ACCTTTGTAGCTGTACCACG | 780bp | 2 |
|  | R:AGCGTGAAGGTAAAACCTGTG |  |  |

**References**

1. Tartof SY, Solberg OD, Manges AR, Riley LW (2005) Analysis of a uropathogenic *Escherichia coli* clonal group by multilocus sequence typing. J Clin Microbiol 43:5860-5864.

2. Nicolas-Chanoine MH, Blanco J, Leflon-Guibout V, Demarty R, Alonso MP, et al. (2008) Intercontinental emergence of *Escherichia coli* clone O25:H4-ST131 producing CTX-M-15. J Antimicrob Chemother 61:273-281.
