# Supplementary material for: IncA/C Plasmid-Mediated Spread of CMY-2 in Multidrug-Resistant Escherichia coli from Food Animals in China
Source: PLoS One. 2014 May 9;9(5):e96738. doi: 10.1371/journal.pone.0096738 (PMC4016023; doi:10.1371/journal.pone.0096738)
Supplement: Table S5 — The number of alleles and ST results for thirty-one CMY-2 producing strains. (DOC) [file pone.0096738.s005.doc]

**Table S5** Allelic profiles and sequence types (STs) assigned in the *Escherichia coli* MLST Database for thirty-one CMY-2 producing strains

| Strains | Multilocus allelic profile^a^ | | | | | | | ST types | ST complex^b^ |
| --- | --- | --- | --- | --- | --- | --- | --- | --- | --- |
|  | adk | fumC | gyrB | icd | mdh | purA | recA |  |  |
| L145 | 10 | 11 | 4 | 8 | 8 | 8 | 2 | ST10 | ST10 C |
| L147 | 10 | 11 | 4 | 8 | 8 | 8 | 2 | ST10 | ST10 C |
| L699 | **6** | 11 | 4 | 8 | 8 | 8 | 2 | ST48 | ST10 C |
| L393 | **6** | 11 | 4 | 8 | **258** | 8 | 2 | ST2690 | ST10 C |
| L78 | **6** | **88** | 4 | 8 | 8 | **6** | 2 | **ST3244** | ST10 C |
| L679 | 43 | 41 | 15 | 18 | 11 | 7 | 6 | ST101 | ST101 C |
| L1119 | 43 | 41 | 15 | 18 | 11 | 7 | 6 | ST101 | ST101 C |
| L669 | 43 | 41 | 15 | **90** | 11 | **8** | 6 | ST359 | ST101 C |
| L670 | 43 | 41 | 15 | **90** | 11 | **8** | 6 | ST359 | ST101 C |
| L671 | 43 | 41 | 15 | **90** | 11 | **8** | 6 | ST359 | ST101 C |
| L667 | 43 | 41 | 15 | **90** | 11 | **1** | 6 | **ST3403** | ST101 C |
| L351 | 6 | 4 | 14 | 16 | 24 | 8 | 14 | ST155 | ST155 C |
| L391 | 6 | 4 | 14 | 16 | 24 | 8 | 14 | ST155 | ST155 C |
| L392 | 6 | 4 | **1** | 16 | 24 | 8 | 14 | ST2294 | ST155 C |
| L361 | 6 | 29 | 32 | 16 | 11 | 8 | 44 | ST156 | none |
| L813 | 6 | 29 | 32 | 16 | 11 | 8 | 44 | ST156 | none |
| L518 | 92 | 4 | 87 | 96 | 70 | 58 | 2 | ST648 | none |
| L461 | 92 | 4 | 87 | 96 | 70 | 58 | 2 | ST648 | none |
| A10-2 | 10 | 27 | 5 | 10 | 12 | 1 | 2 | ST1114 | none |
| T117 | 10 | 27 | 5 | 10 | 12 | 1 | 2 | ST1114 | none |
| L1039 | 101 | 88 | 97 | 108 | 26 | 79 | 2 | ST457 | ST457 C |
| T43 | 101 | 88 | 97 | **25** | 26 | 79 | 2 | **ST3376** | ST457 C |
| L398 | 6 | 65 | 3 | 1 | 11 | 13 | 6 | ST1431 | none |
| L399 | 21 | 35 | 27 | 6 | 5 | 5 | 4 | ST69 | none |
| L215 | 62 | 100 | 17 | 31 | 5 | 5 | 4 | ST362 | none |
| L349 | 85 | 88 | 78 | 29 | 59 | 58 | 62 | ST354 | none |
| L653 | 303 | 41 | 1 | 8 | 8 | 8 | 6 | ST3014 | none |
| T26 | **6** | **11** | **5** | **8** | **7** | **1** | **2** | **ST3404** | none |
| L394 | **6** | **4** | **33** | **16** | **11** | **1** | **6** | **ST3245** | none |
| L1166 | **6** | **438** | **5** | **1** | **8** | **16** | **2** | **ST3269** | none |
| C42 | **8** | **4** | **4** | **8** | **8** | **18** | **6** | **ST3402** | none |

^a^ Allele and ST numbers were those assigned in the *Escherichia coli* MLST database [1]; Novel alleles and sequence types (STs) are in boldface type.

^b^ Each ST founder genotype and its single locus variant (SLV) or double locus variant (DLV) were assigned as a ST complex.

**References:**

[1] *Escherichia coli* MLST database http:// mlst.ucc.ie/mlst/mlst/dbs/Ecoli/
